# Supplementary material for: Effects of different resistance training frequencies on body composition and muscular performance adaptations in men
Source: PeerJ. 2021 Apr 21;9:e10537. doi: 10.7717/peerj.10537 (PMC8067909; doi:10.7717/peerj.10537)
Supplement: Supplemental Information 2 [file peerj-09-10537-s002.docx]

CON: Control Group

RT2: 2 times per week resistance training group

RT4: 4 times per week resistance training group

Pr.Chest Cir: Pre-test for chest circumference

M.Chest.Cir: Mid-test for chest circumference

Po.Chest.Cir: Post-test for chest circumference

Pr.arm.cir: Pre-test for arm circumference

M.arm.cir: Mid-test for arm circumference

Po.arm.cir: Post-test for arm circumference

Pr.thigh.cir: Pre-test for thigh circumference

M.thigh.cir: Mid-test for thigh circumference

Po.thigh.cir: Post-test for thigh circumference

Pr.1RM press: Pre-test for one repetition maximum in bench press

M.1RM press: Mid-test for one repetition maximum in bench press

Po.1RM press: Post-test for one repetition maximum in bench press

Pr.1RM.LP: Pre-test for one repetition maximum in leg press

M.1RM.LP: Mid-test for one repetition maximum in leg press

Po.1RM.LP: Post-test for one repetition maximum in leg press

Pr.End.press: Pre-test for muscle endurance in bench press

M.End.press: Mid-test for muscle endurance in bench press

Po.End.press: Post-test for muscle endurance in bench press

Pr.End.LP: Pre-test for muscle endurance in leg press

M.End.LP: Mid-test for muscle endurance in leg press

Po.End.LP: Post-test for muscle endurance in leg press

Pr.arm curl: Pre-test for arm curl strength

M.arm curl: Mid-test for arm curl strength

Po.arm curl: Post-test for arm curl strength

Pr.VJ: Pre-test for vertical jump

M.VJ: Mid-test for vertical jump

Po.VJ: Post-test for vertical jump

Pr.MBT: Pre-test for medicine ball throw

M.MBT: Mid-test for medicine ball throw

Po.MBT: Post-test for medicine ball throw
